# Adding Help to an HLA-A*24:02 Tumor-Reactive γδTCR Increases Tumor Control

**DOI:** 10.3389/fimmu.2021.752699

**Published:** 2021-10-25

**Authors:** Inez Johanna, Patricia Hernández-López, Sabine Heijhuurs, Wouter Scheper, Laura Bongiovanni, Alain de Bruin, Dennis X. Beringer, Rimke Oostvogels, Trudy Straetemans, Zsolt Sebestyen, Jürgen Kuball

**Affiliations:** ^1^ Center for Translational Immunology, University Medical Center Utrecht, Utrecht, Netherlands; ^2^ Department of Biomolecular Health Sciences, Dutch Molecular Pathology Center, Faculty of Veterinary Medicine, Utrecht University, Utrecht, Netherlands; ^3^ Department of Pediatrics, University Medical Center Groningen, University of Groningen, Groningen, Netherlands; ^4^ Department of Hematology, University Medical Center Utrecht, Utrecht, Netherlands

**Keywords:** cancer immunotherapy, TEGs, mouse model, preclinical (*in vivo*) studies, TCR engineering, human leukocyte antigens (HLA), persistence, efficacy

## Abstract

γδT cell receptors (γδTCRs) recognize a broad range of malignantly transformed cells in mainly a major histocompatibility complex (MHC)-independent manner, making them valuable additions to the engineered immune effector cell therapy that currently focuses primarily on αβTCRs and chimeric antigen receptors (CARs). As an exception to the rule, we have previously identified a γδTCR, which exerts antitumor reactivity against HLA-A*24:02-expressing malignant cells, however without the need for defined HLA-restricted peptides, and without exhibiting any sign of off-target toxicity in humanized HLA-A*24:02 transgenic NSG (NSG-A24:02) mouse models. This particular tumor-HLA-A*24:02-specific Vγ5Vδ1TCR required CD8αα co-receptor for its tumor reactive capacity when introduced into αβT cells engineered to express a defined γδTCR (TEG), referred to as TEG011; thus, it was only active in CD8^+^ TEG011. We subsequently explored the concept of additional redirection of CD4^+^ T cells through co-expression of the human CD8α gene into CD4^+^ and CD8^+^ TEG011 cells, later referred as TEG011_CD8α. Adoptive transfer of TEG011_CD8α cells in humanized HLA-A*24:02 transgenic NSG (NSG-A24:02) mice injected with tumor HLA-A*24:02^+^ cells showed superior tumor control in comparison to TEG011, and to mock control groups. The total percentage of mice with persisting TEG011_CD8α cells, as well as the total number of TEG011_CD8α cells per mice, was significantly improved over time, mainly due to a dominance of CD4^+^CD8^+^ double-positive TEG011_CD8α, which resulted in higher total counts of functional T cells in spleen and bone marrow. We observed that tumor clearance in the bone marrow of TEG011_CD8α-treated mice associated with better human T cell infiltration, which was not observed in the TEG011-treated group. Overall, introduction of transgenic human CD8α receptor on TEG011 improves antitumor reactivity against HLA-A*24:02^+^ tumor cells and further enhances *in vivo* tumor control.

## Introduction

γδT cells share the properties of both innate and adaptive immunity and play an essential role in cancer immunosurveillance (1, 2). Unlike conventional αβT cells, γδT cells recognize their cognate antigens in an MHC-unrestricted manner, targeting stress-induced and malignantly transformed self-antigens (3, 4). As such, γδT cells represent an attractive cell subset to substantiate T cell-based immunotherapeutic strategies that still mainly focus on αβT cells.

Based on their TCRδ chain repertoire, two major subsets of γδT cells can be distinguished: Vδ2^+^ and Vδ2^−^ cells. Vδ2^+^ cells mainly reside in the human peripheral blood, representing up to 5% of total circulating T cells, and sense metabolic changes in tumor cells with intracellular accumulation of phosphoantigens (pAgs) level. Vδ2^+^ T cell recognition is facilitated by butyrophilin (BTN) family molecules, including BTN2A1 and BTN3A1 (5–10). On the other hand, Vδ2^−^ cells mainly localize in mucosal and epithelial tissues, but their antitumor properties are scarcely known (4). Vδ2^−^ cells recognize a broad range of stress-induced ligands, such as the MHC-associated proteins MICA and MICB, foreign lipid antigens presented on CD1c/d molecules in classical HLA-like manner, and CMV-associated UL16-binding protein (ULBP) family members, that are upregulated in stressed or malignant cells (11–15).

Vδ1^+^ T cells, one of the major Vδ2^−^ subsets, have been shown to exert antitumor reactivity against leukemia and solid tumors (16–21), indicating their potential in cancer immunotherapy. Adoptive transfer of *in vitro* expanded Vδ2^+^ cells only showed marginal clinical responses to date (4, 22), while adoptive transfer of Vδ2^−^ cells is yet to be tested in the clinic (23). Translational efforts using γδT cells and their receptors outside the context of allogeneic stem cell transplantation (24, 25) face substantial hurdles, due to their limited proliferative capacity, underestimated diversity in co-receptors expression and function, as well as scarce information on how γδTCRs interact with their targets.

To bypass these major drawbacks of translating γδT cells-based immune therapies into clinical practice, we developed the concept of TEGs: αβT cells engineered to express a defined γδTCR, allowing the introduction of highly tumor-reactive γδTCR, both Vδ2^+^ (26, 27) or Vδ2^−^ (28, 29) subsets, into proliferatively-proficient αβT cells (27, 30, 31). This concept did not only allow to select for highly tumor-reactive γδTCR, but also within the context of Vδ2^+^ TCRs to reprogram both CD4^+^ and CD8^+^ αβT cells (26, 27). Professional help for TCR-engineered CD8^+^ αβT cells by also functionally engineering CD4^+^ αβT cells has not only been shown to be important *in vitro* (32) but also to improve clinical responses (33). Within this context, we previously identified an allo-HLA-restricted and tumor-specific Vγ5Vδ1TCR derived from clone FE11, introduced in the TEG concept as TEG011, which was, although not dependent on a defined peptide, selectively targeting HLA-A*24:02^+^ tumor cells without impairing the healthy tissues (34). Furthermore, we also highlighted that antitumor reactivity of Vγ5Vδ1TCR derived from clone FE11 requires CD8α as costimulatory receptor and showed that both CD8αα on the original clone FE11 and CD8αβ on transduced αβT cells are capable of providing costimulation to the Vγ5Vδ1TCR derived from clone FE11 (34). Thus, for this very particular Vγ5Vδ1TCR, the concept of TEGs would not benefit from reprogramming CD4^+^ αβT cells when only a Vγ5Vδ1TCR is transferred as CD4-transduced TEG011 cells do not elicit antitumor reactivity.

Human CD8 is a membrane glycoprotein classified in an immunoglobulin-like superfamily consisting of hetero- or homodimer of α and β chains, making up for the CD8αβ or CD8αα co-receptor on the cell surface. CD8αβ predominantly expressed on αβT cells, while CD8αα mainly expressed on the cell membrane of innate immune cells, including macrophages, dendritic cells, natural killer (NK) cells, and γδT cells (35). Transfer of CD8 receptor has been reported for αβTCR engineered αβT cells to functionally reprogram CD4^+^ αβT cells, when low to intermediate affinity αβTCRs are used for engineering (36). Within this context, we addressed the implication of CD8αα-dependency of FE11 γδTCR in relation to its tumor immunity. Based on this mechanistic basis of antitumor reactivity for TEG011 cells, we hypothesize that the transfer of CD8α receptor can functionally rescue Vγ5Vδ1TCR engineered CD4^+^ αβT cells. Within this context, we explored now as additional approach to improve the efficacy of TEG011 therapy, the simultaneously co-expressing Vγ5Vδ1TCR derived from clone FE11 together with CD8α receptor in a TEG format, referred to as TEG011_CD8α. Importantly, we demonstrate that introduction of transgenic human CD8α co-receptor into CD4^+^ TEG011 cells successfully enhanced its antitumor efficacy *in vitro* and *in vivo*, and thus did not require CD8β. Furthermore, we show that the co-expression of CD8α in CD4^+^ TEG011 provides additional survival signal and facilitates better T-cell persistence and infiltration *in vivo*, both of which are essential to sustain long-term tumor control of adoptively transferred TCR-based immunotherapy.

## Materials and Methods

### Cell Lines

Daudi, SW480, and Phoenix-Ampho cell lines were obtained from ATCC. K562 with HLA-A*24:02-transduced cell line was kindly provided by Fred Falkenburg (Leiden University Medical Centre, Netherlands) and subsequently transduced with luciferase for *in vivo* imaging purposes. EBV-LCL was kindly provided by Phil Greenberg (Seattle, WA, USA). Phoenix-Ampho and SW480 cells were cultured in DMEM supplemented with 1% Pen/Strep (Invitrogen) and 10% FCS (Bodinco), whereas all other cell lines in RPMI with 1% Pen/Strep and 10% FCS. All cell lines were authenticated by short tandem repeat profiling/karyotyping/isoenzyme analysis and were passaged for a maximum of 2 months, after which new cell line stocks were thawed for experimental use. Furthermore, all cell lines were routinely verified by growth rate, morphology, and/or flow cytometry and tested negative for mycoplasma using MycoAlert Mycoplasma Kit (Lonza, Breda, Netherlands). Peripheral blood mononuclear cells (PBMCs) from healthy donors were isolated by Ficoll-Paque (GE Healthcare, Eindhoven, Netherlands) from buffy coats supplied by Sanquin Blood Bank (Amsterdam, Netherlands).

### Cloning of TEG011_CD8α and TEGLM1_CD8α

Clone FE11 was generated as previously described (28). FE11 and LM1 [non-functional γ9δ2TCR with length mutation on the complementary determining region 3 (CDR3) of the δ2-chain (31)] γδTCRs were subcloned to pMP71 retroviral vectors containing both γTCR and δTCR chains, separated by a ribosomal skipping T2A sequence. pU57 constructs containing a ribosomal skipping P2A sequence, followed by full-length human CD8α, were purchased from Baseclear (Leiden, Netherlands). Thereafter, CD8α was subcloned into pMP71 vector using *Xho*I and *Hind*III restriction sites downstream of γ115TCR-T2A-δ115_LM1 sequence to generate a TEGLM1_CD8α (**Supplementary Table 2**) construct that contained *Nco*I and *Xho*I restriction sites up- and downstream of LM1 γδTCR chains. *Nco*I and *Xho*I restriction sites were then inserted up- and downstream of FE11 γδTCR sequences by site-directed mutagenesis PCR, after which this sequence was ligated to P2A-CD8α sequence in pMP71 vector using the introduced *Nco*I and *Xho*I sites, generating a TEG011_CD8α construct (**Supplementary Table 1**). Where indicated, CD4^+^, CD8^+^, CD4^+^CD8αα^+^, and CD4^+^CD8αβ^+^ TCR-transduced T cells were sorted using a FACSAria II (BD) flow cytometry to >99% purity. Expression levels of CD8α mutants were measured by flow cytometry using anti-CD8α antibody (clones RPA-T8).

### Functional T-Cell Assays

IFNγ ELISPOT was performed using antihuman IFNγ mAb1-D1K (I) and mAb7-B6–1 (II) (Mabtech) per the manufacturer’s protocol. Then 15,000 TEG cells (TEG011, TEGLM1, TEG011_CD8α, or TEGLM1_CD8α) were co-incubated with 50,000 target cells (E:T ratio 1:3) for 18–24 h in nitrocellulose-bottomed 96-well plates (Millipore). IFNγ spots were visualized with TMB substrate (Sanquin), and subsequently the number of spots was quantified using ELISPOT Analysis Software (Aelvis). Where indicated, blocking of CD8α was performed using 10 μg/ml anti-CD8α antibody clone OKT8 (eBioscience) and blocking of CD8β with 10 μg/ml anti-CD8β clone 2ST8.5H7 (Abcam).

### Retroviral Transductions of T Cells

TEGs were generated as previously described (30). Briefly, Phoenix-Ampho packaging cells were transfected with gag-pol (pHIT60), env (pCOLT-GALV), and pMP71 retroviral constructs containing both γTCR and δTCR chains separated by a ribosomal skipping T2A sequence and followed by CD8α sequence separated by P2A sequence where applicable, using FugeneHD reagent (Promega, Leiden, Netherlands). PBMCs from a healthy donor preactivated with 30 ng/ml anti-CD3 (clone OKT3, Miltenyi Biotec) and 50 IU/ml IL-2 (Proleukin, Novartis, Arnhem, Netherlands) were transduced twice with viral supernatant within 48 h, in the presence of 50 IU/ml IL-2 and 6 µg/ml polybrene (Sigma-Aldrich, Zwijndrecht, Netherlands). TCR-transduced T cells were expanded by stimulation with anti-CD3/CD28 Dynabeads (500,000 beads/10^6^ cells; Thermo Fisher Scientific, Breda, Netherlands) and 50 IU/ml IL-2. Thereafter, transduced T cells were depleted of the non-engineered T cells.

### Depletion of Non-Engineered T Cells

Non-engineered T cells were depleted as previously described (27). In brief, transduced T cells were incubated with a biotin-labeled anti-αβTCR antibody (clone BW242/412; Miltenyi Biotec, Leiden, Netherlands) and then incubated with an anti-biotin antibody coupled to magnetic beads (anti-biotin MicroBeads; Miltenyi Biotec), most recently reported to preferentially bind to the βTCR chain (37). Thereafter, the cell suspension was loaded onto an LD column, and αβTCR^+^ T cells were depleted by MACS cell separation per the manufacturer’s protocol (Miltenyi Biotec). After depletion, TEGs were expanded using a T-cell rapid expansion protocol (REP) (30).

### Separation of CD4^+^ Subsets of TEGs

The separation of CD4^+^ TEGs was performed using CD4 Microbeads (Miltenyi Biotech) as per the manufacturer’s instructions. Briefly, TEGs that were previously expanded on REP were incubated with magnetic microbeads cells and loaded into LS column for MACS cell separation. Thereafter, CD4^+^ selected or bulk (with CD4:CD8 ratio 50:50) TEGs were expanded separately on the next REP cycle prior to *in vitro* functional assay. TEG expression was monitored prior to functional assays or *in vivo* infusion by flow cytometry using anti-αβTCR-APC (clone IP26, eBioscience), anti-pan-γδTCR-PE (clone IMMU510, Beckman Coulter), anti-CD8-PerCP-Cy5.5 (clone RPA-T8, Biolegend), anti-CD4-PeCy7 (clone TPA-R4, Biolegend), anti-CD4-FITC (clone TPA-R4, Biolegend), and Vδ1-FITC (clone TS8.2, Thermo Fisher Scientific) antibodies.

### Animal Model

The NOD.Cg-*Prkdc^scid^Il2rg^tm1Wjl^* Tg(HLA-A24)3Dvs/Sz (NSG-A24:02) mice (38) were bred and housed in the breeding unit of the Central Animal Facility of Utrecht University. Experiments were conducted per institutional guidelines after obtaining permission from the local ethical committee, and performed in accordance with the current Dutch laws on animal experimentation. Mice were housed in individually ventilated cage (IVC) system to maintain sterile conditions and fed with sterile food and water. After irradiation, mice were given the antibiotic ciproxin in the sterile water throughout the duration of the experiment. Both male and female mice were randomized with equal distribution among the different groups, based on age and initial weight (measure on Day −1) into 10 mice/group. Adult NSG-A24:02 mice (11–20 weeks old) received sublethal total body irradiation (1,75 Gy) on day −1 followed by intravenous injection of 1×10^5^ K562-HLA-A*24:02 luciferase tumor cells on day 0, and received 2 intravenous injections of TEG011, TEG011_CD8α, or TEGLM1_CD8α cells on days 1 and 6 as previously reported (34). Together with the first TEGs injection, all mice received 0,6 × 10^6^ IU of IL-2 (Proleukin; Novartis) in 100 µl incomplete Freund’s adjuvant (IFA) subcutaneously and subsequently administered every 3 weeks until the end of the experiment. Mice were monitored at least twice a week for any symptoms of disease (sign of paralysis, weakness, and reduced motility), weight loss, and clinical appearance scoring (scoring parameter included hunched appearance, activity, fur texture, and piloerection). The humane endpoint was reached when mice showed the aforementioned symptoms of disease, experienced a 20% weight loss from the initial weight (measured on day −1), developed extramedullary solid tumor masses (if any) reached 2 cm³ in volume, and when clinical appearance score 2 was reached for an individual parameter or a total score of 4.

### Flow Cytometry Analysis

The following antibodies were used for flow cytometry analysis: huCD45-PB (clone HI30; Sony Biotechnology), pan-γδTCR-PE (clone IMMU510; Beckman-Coulter), mCD45-APC (clone 30-F11, Sony Biotechnology), αβTCR-FITC (clone IP26; Biolegend), CD4-PeCy7 (clone RPA-T4, Biolegend), CD8-PerCPCy5.5 (clone RPA-T8, Biolegend), PD-1-BV711 (clone EH12.2H7, Biolegend), and TIM3-BV650 (clone F38-2E2, Biolegend). To exclude non-viable cells from the analysis, Fixable Viability Dye eFluor506 was used (eBioscience). All samples were analyzed on a BD LSRFortessa using FACSDiva Software (BD Biosciences).

### Assessment for TEGs Persistence

Mouse peripheral blood samples were obtained *via* cheek vein (max. 50–70 µl/mouse) once a week. Red blood cells were lysed using 1× RBC lysis buffer (Biolegend) and were then stained with a mixture of antibody panels as listed above. The persistence of TEG cells was counted as absolute cell number tumor-reactive TEG cells expressing following cell surface markers huCD45^+^γδTCR^+^CD8^+^ and huCD45^+^γδTCR^+^CD4^+^CD8^+^ populations or non-reactive TEG cells expressing huCD45^+^γδTCR^+^CD4^+^ marker observed in mouse peripheral blood using Flow-count Fluorospheres (Beckman Coulter) and measured by flow cytometry.

### Preparation of Single-Cell Suspensions

At the end of the study period, bone marrow (mixed from tibia and femur) and spleen sections were isolated and processed into single-cell suspension. Femur and tibia from the hind legs were collected; bone marrow cells were collected by centrifugation of the bones at 10,000 rpm for 15 s and resuspension of the cells in phosphate buffer saline (PBS).

A small section of the spleen was minced and filtered through a 70 µm cell strainer (BD); incubated with 1× RBC lysis buffer cells for maximum 4 min, and subsequently cells were washed and resuspended in PBS.

Absolute cell number of TEG cells were quantified using Flow-count Fluorospheres and measured from a total of 10^6^ cells stained for the presence of TEG cells in spleen and bone marrow by flow cytometry analysis (BD LSRFortessa).

### Histology Staining and Analysis

Formalin-fixed femur for bone marrow sections were embedded in paraffin and cut into 4 μm sections. Hematoxylin and eosin (H&E) staining was performed for the femur, for bone marrow section. Tissue sections were evaluated to assess for any differences in the presence, distribution, and extension of neoplastic foci indicating tumor tissue. Tissue sections of the femur were evaluated for quantification of tumor tissue by dividing the area covered by the tumor cells by the total area of bone marrow tissue visible in the section using the ImageJ analysis system software (NHI, Bethesda, Maryland, USA) and expressed as a percentage. Images were taken using an Olympus BX45 microscope with the Olympus DP25 camera and analyzed using DP2-BSW (version 2.2) or ImageJ software.

### Statistical Analyses

Experimental data were analyzed using GraphPad Prism (GraphPad Software Inc., La Jolla, CA, USA) and shown as mean ± standard deviation (SD) or standard error of mean (SEM) with *P < 0.05; **P < 0.01; ***P < 0.001; and ****P < 0.0001. Statistical significances between groups were assessed using a non-parametric Kruskal-Wallis test, a two-way ANOVA, and a mixed-effects model with repeated measures where indicated.

## Results

### Co-Transfer of Transgenic CD8α Receptor Is Sufficient to Re-Establish Tumor Reactivity of CD4^+^ TEG011 Cells

**Figure 1:**
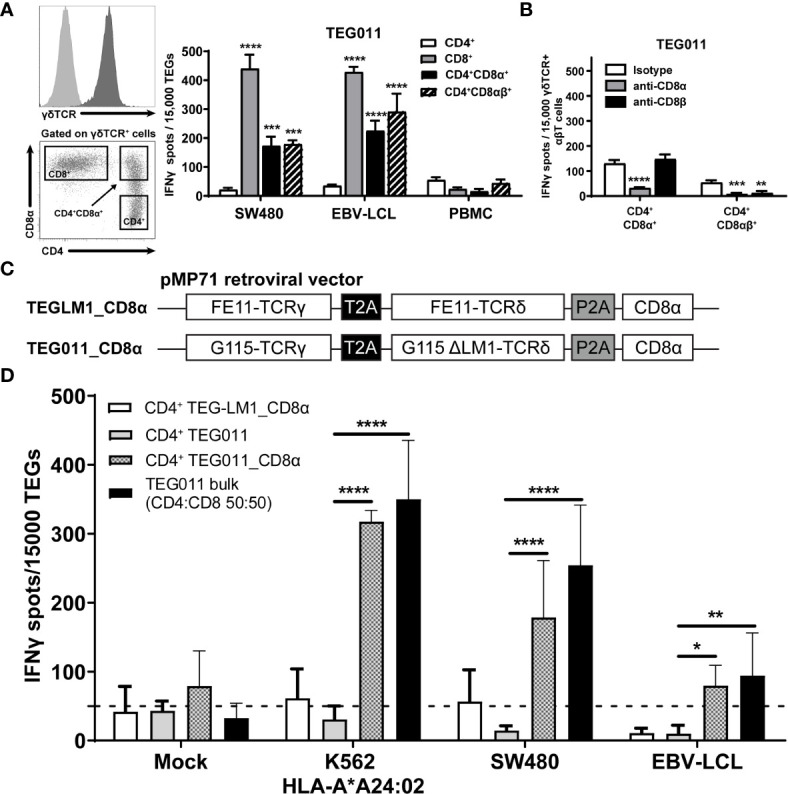
Introduction of transgenic CD8α receptor on TEG011 improves T cell activation. **(A)** TEG011 were retrovirally transduced with either CD8α alone or CD8α in combination with CD8β. CD4^+^, CD8^+^, CD4^+^CD8α^+^, and CD4^+^CD8αβ^+^ subsets of T cells were subsequently sorted (left panel is a representative sorting plot for CD4^+^, CD8^+^, and CD4^+^CD8α^+^ cells; CD4^+^CD8αβ^+^ cells were sorted in a similar manner) and tested for recognition of SW480 and EBV-LCL target cells by IFNγ ELISPOT (right panel). Healthy PBMCs were included as untransformed mock control target cells. Data are of representative of four independent experiments, and error bars represent mean ± SEM (**P < 0.01; ***P < 0.001) calculated by two-way ANOVA. **(B)** CD8α and CD8β blocking on CD4^+^ T cells were transduced with the FE11 γδTCR and CD8α alone, or CD8α with CD8β. TEG011 was co-incubated with SW480 target cells in the presence of a control antibody, or CD8α or CD8β blocking antibodies. IFNγ production was measured by ELISPOT. Data represent mean ± SD of replicates for each effector (**P < 0.01; ***P < 0.001; ****P < 0.0001) calculated by two-way ANOVA. **(C)** Schematic diagram of pMP71 retroviral vector constructs containing codon-optimized human γδTCR sequences from either clone FE11 (referred as TEG011_CD8α) or non-functional LM1 chains (referred as TEGLM1_CD8α) in combination with full length of human CD8α receptor (top panel). Within the transgene cassettes, individual γTCR and δTCR chains have been linked with a self-cleaving thosea asigna virus 2A (T2A; black box) ribosomal skipping sequence, while the CD8α sequence was connected with a porcine teschovirus-1–derived 2A (P2A; gray box) ribosomal skipping sequence. **(D)** CD4^+^ αβT cells were transduced with either TEGLM1_CD8α, TEG011, or TEG011_CD8α γδTCR (as effector cells) and subsequently co-cultured with HLA-A*24:02-expressing target cell lines or healthy T cells (E:T ratio is 1:3) for 18–24 h. TEG011 bulk population with 50:50 ratio of both CD4^+^ and CD8^+^ TEGs and T cells from healthy donor were used as positive and untransformed mock controls, respectively. Antitumor reactivity was measured by IFNγ ELISPOT, where 50 spots/15,000 cells were considered as a positive antitumor response and indicated by the dashed horizontal line. Data are representative of three independent experiments with replicates for each target, and error bars represent mean ± SD (*P < 0.05; **P < 0.01; ****P < 0.0001) calculated by two-way ANOVA.

We previously identified an allo-restricted CD8α-dependent Vγ5Vδ1TCR clone FE11 (28), which showed *in vitro* antitumor reactivity against HLA-A*24:02-expressing tumor cells (34). We therefore investigated whether introduction of CD8αα or CD8αβ along with Vγ5Vδ1TCR derived from clone FE11 could enhance antitumor reactivity of CD8^+^, and also functionally reprogram CD4^+^ TEG011 cells. Hence, we co-transduced T cells with the FE11 γδTCR, and with either CD8α alone or CD8α together with CD8β (**Figure S1**). Subsequently, we sorted separate sets of CD4^+^ TEG011 cells that co-expressed either exogenous CD8αα (CD4^+^CD8α^+^) or CD8αβ (CD4^+^CD8αβ^+^) as well as TEG011 cells expressing only endogenous CD4 and CD8 as negative and positive controls for tumor recognition, respectively (**Figure 1A**). Thereafter, TEG cells were co-cultured with SW480 and EBV-LCL target cells or healthy PBMCs as mock control. Both CD4^+^CD8α^+^ and CD4^+^CD8αβ^+^ TEG011 cells secreted significantly higher levels of IFNγ upon exposure to tumor targets than CD4^+^ TEG011 cells. The acquired antitumor reactivity of CD4^+^CD8α^+^ and CD4^+^CD8αβ^+^ TEG011 cells could be blocked by CD8α and CD8β blocking antibodies (**Figure 1B**), confirming the strict dependence of FE11 γδTCR on introduced CD8 molecules. Taken together, we showed that introduction of CD8α alone is sufficient to re-establish antitumor reactivity of CD4^+^ T cells expressing FE11 γδTCR. Introduction of CD8β did not further enhance tumor recognition but was functionally involved in the molecular interaction with its target when present.

For clinical administration, co-expression of both CD8α and the γδTCR in one vector is preferred to allow reproducible and cost-effective production processes (26, 27, 39). Moreover, co-expressing both CD8α and the γδTCR in one vector can also overcome the difference in transduction efficiency when they were transduced separately. Therefore, we generated new retroviral constructs carrying either FE11 γδTCR or a non-functional length mutant clone LM1 γδTCR [ (31); served as mock control] followed by full-length human CD8α receptor sequences (TEG011_CD8α and TEGLM1_CD8α, **Figure 1C**). The complete sequence of transgenes for these retroviral constructs is listed in **Supplementary Tables 1, 2**, respectively. Subsequently, αβT cells were transduced with either FE11 γδTCR without human CD8α receptor (TEG011), FE11 γδTCR with human CD8α receptor (TEG011_CD8α), or LM1 γδTCR with human CD8α receptor (TEGLM1_CD8α). After TEG expansion, we performed magnetic selection of CD4^+^ T cells for each TEG constructs. To elucidate whether introduction of transgenic CD8α receptor adequately rescues TEG011 reactivity of non-tumor reactive CD4-transduced cells once delivered by the very same vector, we co-cultured tumor target HLA-A*24:02-transduced CML tumor cells (K562), SW480, and EBV-LVL cells with either CD4^+^ TEG011_CD8α, CD4^+^ TEGLM1_CD8α, or CD4^+^ TEG011 (without introduction of the CD8α receptor). Healthy T cells and TEG011 bulk cells (with CD4:CD8 1:1 ratio) were used as the untransformed mock target and positive effector control, respectively (**Figure 1D**). CD4^+^ TEG011_CD8α cells produced a significantly higher IFNγ level compared to CD4^+^ TEG011, which was equivalent to those of TEG011 bulk cells against all tumor targets, without affecting healthy cells. The equivalent IFNγ level between CD4^+^ TEG011_CD8α and TEG011 bulk cells comprised of only 50% CD8^+^ TEG011 implied that reprogrammed CD4^+^ TEG011_CD8α are surprisingly poorer cytokine secretors. Importantly, enhanced tumor recognition was restricted to CD4^+^ TEG011_CD8α cells and not CD4^+^ TEGLM1_CD8α mock cells, highlighting the specific role of CD8α as co-stimulation for the introduced FE11 γδTCR. We concluded that introduction of transgenic CD8α receptor in combination with Vγ5Vδ1TCR derived from clone FE11 allowed reprogramming of CD4^+^ T cells towards HLA-A*24:02-expressing tumor cells *in vitro*, though activity was lower when compared to CD8^+^ TEG011.

### TEG011_CD8α Improves *In Vivo* Tumor Control and Associates With Higher Persistence of Functional T Cells

**Figure 2:**
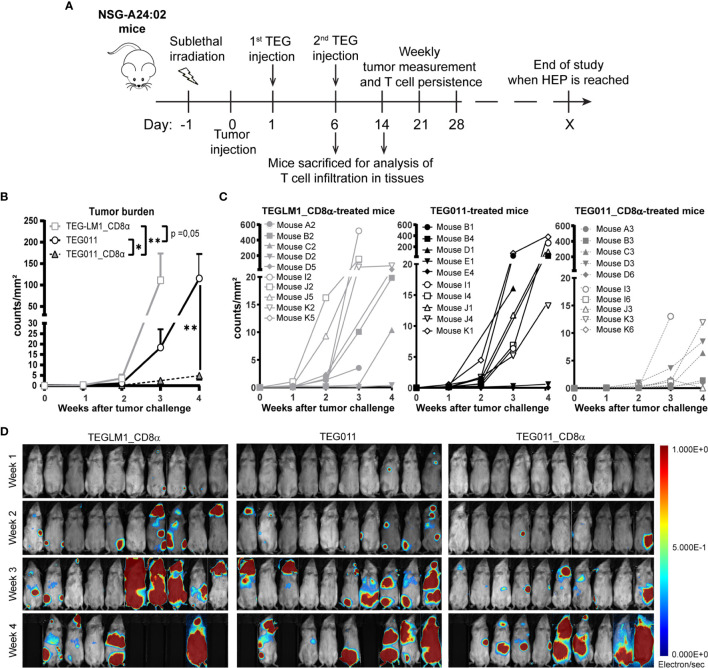
TEG011_CD8α improves *in vivo* tumor control against HLA-A*24:02^+^ tumor cells. **(A)** Schematic overview of the *in vivo* experiment for NSG-A24:02 tumor-bearing mice. Irradiated mice were intravenously injected with K562-HLA*A24:02-luciferase tumor cells on day 0 followed by two infusions of TEG011, TEG011_CD8α, or TEGLM1_CD8α mock cells on days 1 and 6. Mice were monitored regularly and sacrificed when the humane endpoint (HEP) was reached. **(B)** Tumor burden for K562-HLA*A24:02-luciferase was assessed *in vivo* measuring integrated signal density per total surface area (count/mm^2^) by bioluminescence imaging (BLI) with the mouse abdomen facing up. Data are shown only up to week 3 for the TEGLM1_CD8α mock-treated group (open light gray rectangle) due to subsequent mouse dropout >50%, while data for TEG011 (open black circle) and TEG011_CD8α (open black triangle) are shown up to week 4. Data are shown as mean ± SEM of all mice per group (n = 10). Statistical significances were calculated by a mixed-effects model with repeated measure up to week 3 as comparison all treatment group (indicated next to legends) and only between TEG011 and TEG011_CD8α group for week 4 (indicated on the graph); (*P < 0.05; **P < 0.01). **(C)** Tumor burden for individual mouse for each treatment group measured by integrated signal density per total surface area (count/mm^2^) using BLI. **(D)** Tumor load for individual mouse was evaluated by bioluminescence imaging on week 1 to week 4 using Milabs Optical Imaging (OI) Acquisition and OI-Post processing software (version 2.0). Anesthetized mice were injected intraperitoneally with 25 mg/ml Beetle-luciferin (Promega). Calibrated units were calculated from integrated density of bioluminescence signal (electron/s) as shown by the right bar. The animals were imaged 10 min after luciferin injection. Black areas indicate loss of mice.

In previous studies, we have shown TEG011 efficacy against HLA-A*24:02-expressing tumor cells *in vitro* and an extended *in vivo* safety profile, as well as peripheral persistence of TEG011, where long-term persistence of TEG associated with reduced probability for developing extramedullary solid tumor masses *in vivo* (34, 40). To assess the consequence of the additional expression of TEG011_CD8α, NSG transgenic mice expressing human HLA-A*24:02 (NSG-A24:02) were irradiated, received luciferase-labeled K562 HLA-A*24:02^+^ cells, and subsequently received two intravenous injections of either mock control TEGLM1_CD8α, TEG011_CD8α, or TEG011 cells. All infused TEG variants showed comparable γδTCR expression, where the transduced αβT cells expressed Vδ1^+^ TCR for TEG011 and TEG011_CD8α (**Figure S2**). Mice were monitored for tumor burden assessed by bioluminescent imaging, T cell persistence and infiltration, as well as any other signs of discomfort. Mice were sacrificed when the humane endpoints were reached (experimental outline **Figure 2A**). TEG011_CD8α-treated mice had a significantly lower tumor burden over time compared to the mock control TEGLM1_CD8α and TEG011-treated groups (**Figure 2B**), indicating superior tumor control *in vivo* by TEG011_CD8α. All tumor-bearing mice eventually developed tumor, and measurement of individual mouse indicating tumor growth over time for each treatment group is shown in **Figures 2C, D**. Despite the significant *in vivo* tumor control, we observed only a trend towards an improved overall survival for TEG011_CD8α-treated mice (**Figure S3**). This could be due to limited treatment window of this mouse model contributed by aggressive tumor growth of K562 HLA-A*24:02-transduced cells.

**Figure 3:**
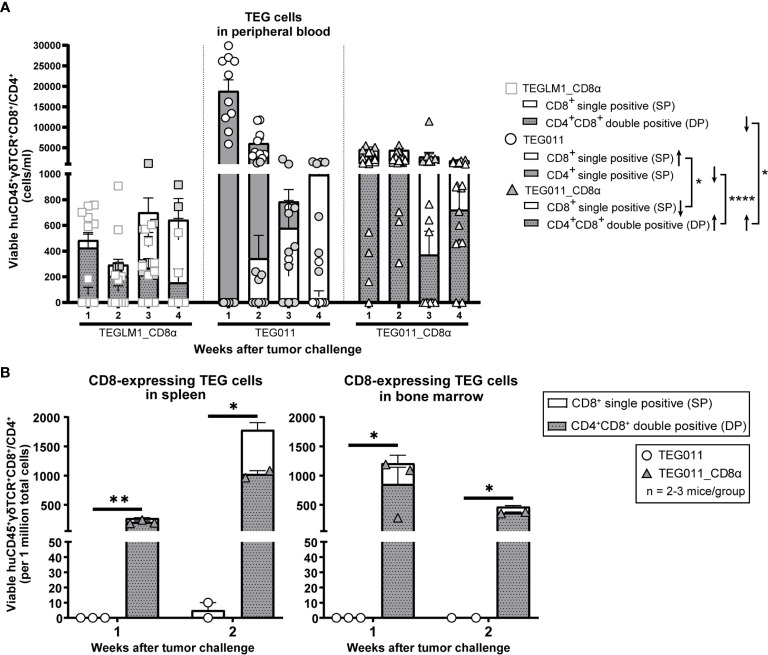
TEG011_CD8α enhances TEG persistence and infiltration. **(A)** TEG cells were measured in peripheral blood using flow cytometry by quantifying the absolute cell numbers of TEGLM1_CD8α mock (open light gray rectangle), TEG011 (open black circle), and TEG011_CD8α (open black triangle) in tumor-bearing mice. TEG cells are distinguished into different cellular compartments: CD8^+^ single-positive (SP; white stacked bar), CD4^+^ single-positive (SP; gray stacked bar), and CD4^+^CD8^+^ double-positive (DP; gray dotted stacked bar) cells. Black arrows indicate higher or lower T cell counts observed. Data are shown as mean ± SEM of all mice per group (n = 10 mice). Statistical significances were calculated by a mixed-effects model with repeated measures (*P < 0.05; ****P < 0.0001). **(B)** CD8-expressing TEG cells was assessed in spleen and bone marrow by quantifying the total viable cells of huCD45^+^γδTCR^+^CD8^+^ and huCD45^+^γδTCR^+^CD4^+^CD8^+^ per one million single-cell suspension by flow cytometry. Cell counts of individual mouse per treatment group are represented by each symbol. Functional TEG011 cells consist of two different cellular compartments: CD8^+^ single-positive (SP; white stacked bar) and CD4^+^CD8^+^ double-positive (DP; gray dotted stacked bar). Data are shown as mean ± SEM (*P < 0.05; **P < 0.01) calculated by a mixed-effects model with repeated measures.

As TEG011 cells carry CD8α-dependent Vγ5Vδ1TCR, we focused our *in vivo* analysis to tumor-reactive CD8-expressing TEG cells (as validated by *in vitro* functional T cell assay in **Figure 1D**) while taking into account the non-tumor reactive CD4^+^ TEG cells. Therefore, we assessed CD8-expressing TEG cell product properties and persistence by measuring viable huCD45^+^γδTCR^+^CD8^+^ single-positive and huCD45^+^γδTCR^+^CD4^+^CD8^+^ double-positive cells (present in mock control TEGLM1_CD8α and TEG011_CD8α only) in mouse peripheral blood using flow cytometry (gating strategy depicted in **Figure S4**). TEG cells persisted up to 4 weeks after infusion in the mouse peripheral blood with biological variations between mice (**Figure 3A**). To address this interindividual variation in T-cell persistence, we analyzed separately the percentage of mice where CD4^+^ and CD8^+^ T cells reached at least 500 cells/ml in the peripheral blood over time, a threshold described previously (41) (**Figure S5A**). We observed a higher percentage of mice with persisting CD4^+^ and CD8^+^ T cells in TEG011_CD8α group when compared to mock TEGLM1_CD8α and TEG011 group. Despite some imbalance in the CD4:CD8 ratio with lower numbers for CD8^+^ TEG011 infused (**Figure S2**), more CD8^+^ TEG011 persisted over time when compared to CD8^+^ single-positive TEG011_CD8α. *Vice versa*, endogenous CD4 T cells for TEG011_CD8α were lower before infusion when compared to TEG011 prior to infusion, while CD4^+^CD8^+^ double-positive TEG011_CD8α were higher in numbers over time when compared to both CD4^+^CD8^+^ double-positive TEGLM1_CD8α and CD4^+^ TEG011 cells (**Figure S5B**). As a net effect, we observed more CD8-expressing T cells for TEG011_CD8α cells when compared to TEG011 (**Figure 3B**). Next, we investigated the expression of PD1 and TIM3 on CD8^+^ single-positive cells and CD4^+^ single-positive or CD4^+^CD8^+^ double-positive cells. Higher numbers of T cells expressing PD1 or TIM3 were observed on TEG011_CD8α cells, as compared to mock TEGLM1_CD8α and TEG011 cells (**Figures S6A, B**). CD8^+^ single-positive TEG011 and TEG011_CD8α showed an increased PD1 expression when compared to CD8^+^ single-positive TEG_LM1 (**Figure S6A**). A partial decline of TIM3 expression was most pronounced over time in CD8^+^ single-positive TEG011_CD8α (**Figure S6B**).

Next, we investigated infiltration of TEG cells into spleen and bone marrow on weeks 1 and 2 after infusion. Specifically, we compared the TEG011 and TEG011_CD8α groups to elucidate the contribution of transgenic CD8α co-expression in TEG011 infiltration *in vivo*, and focused on the total sum of CD8-expressing TEG011 cells. We detected a significantly higher number of CD8-expressing TEG cells infiltrating in the spleen and bone marrow of TEG011_CD8α-treated mice at both time points (**Figure 3B**). Importantly, we did not observe rapid clearance of CD4^+^CD8^+^ double-positive TEG011_CD8α cells in these tissues within these time points, whereas CD8^+^ single-positive TEG011 cells were barely detected. Thus, we conclude that CD8α co-stimulation with TEG011 improves overall *in vivo* tumor control, T cell persistence, and infiltration of CD8-expressing TEG011 cells.

### TEG011_CD8α Enhanced T Cell Infiltration and Effectively Cleared Tumor Cells in Bone Marrow

**Figure 4:**
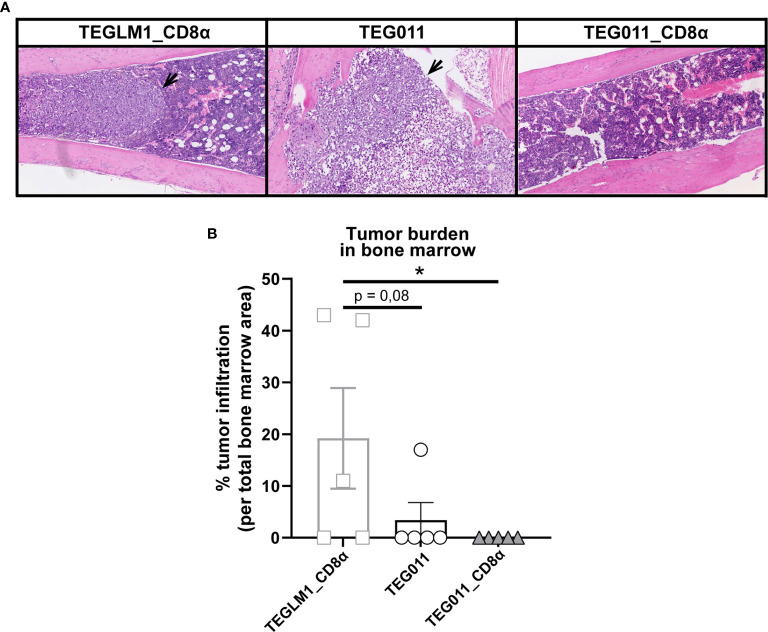
TEG011_CD8α effectively cleared tumor cells in bone marrow, without a significant difference in tumor infiltration observed in other major organs. **(A)** Representative pictures H&E stained of mouse bone marrow with the presence of neoplastic cells (black arrow) from individual mice of each treatment group (n = 5 mice/group). Magnification: 10×. **(B)** Percentage cases of tumor infiltration in mouse bone marrow for each treatment group (n = 5 mice/group). Calculation was performed by dividing the area covered by the tumor cells per the total area of bone marrow tissue visible in the section using ImageJ. Data are shown as mean ± SEM (*P < 0.05) calculated by non-parametric Kruskal-Wallis test.

We previously reported an extensive *in vivo* safety profile of TEG011 against healthy tissues that express HLA-A*24:02 molecules, in which no significant histological lesions were observed in major organs, including liver, spleen, and intestine (40). For histopathology analysis, we collected a femur bone marrow section from each treatment group at the end of the study period to further evaluate antitumor efficacy of the new TEG011_CD8α cells (**Figure 4A**). Tissue sections were assessed for the presence and extension of the neoplastic foci composed by round, large, undifferentiated tumor cells. The mock control TEGLM1_CD8α-treated group showed evident 19,2% neoplastic infiltration, whereas the TEG011-treated group showed up to 3,4% neoplastic infiltration of a homogeneous population of neoplastic cells in the bone marrow. Interestingly, we did not observe any neoplastic infiltration in the bone marrow of mice in the TEG011_CD8α group, and the appearance of bone marrow cell composition and cellularity was normal (**Figure 4B**). In conclusion, although the number of analyzed bone marrows was limited, our data imply that TEG011_CD8α effectively cleared tumor cells in bone marrow, emphasizing the role of CD8α co-stimulation for better *in vivo* tumor control of TEG011 cells. Overall, our data indicate that introduction of transgenic CD8α on TEG011 cells effectively improves *in vivo* tumor control and better T cell infiltration into bone marrow.

## Discussion

TEG011 has been reported to specifically recognize HLA-A*24:02^+^ malignant cells while sparing the HLA-A*24:02-expressing healthy tissues with the requirement of CD8α co-stimulation (34, 40). While TEG011 has shown a favorable efficacy profile *in vivo*, we only observed in approximately 50% of the mice long-term persistence of CD8^+^ TEG011 cells, which could be due to the lack of support by antigen-specific CD4^+^ T cells (29, 40). The presence of both tumor-specific CD4^+^ and CD8^+^ αβT cells has been reported to significantly improve clinical responses compared to tumor-specific CD8^+^ αβT cells alone (33). To further improve the antitumor efficacy of TEG011, we co-expressed a CD8α co-receptor together with the Vγ5Vδ1TCR derived from clone FE11 in TEG format, referred to as TEG011_CD8α cells. Introduction of CD8α receptor is particularly beneficial for TEG011 as this particular γδTCR requires the presence of CD8α as co-receptor for their antitumor reactivity, as we published previously (34, 40). CD8α expression has been reported as common feature of γδTCR after CMV infection (28). These insights imply that also other Vδ1TCR might functionally depend on CD8α, which we could, however, not investigate in a broader context. Thus, when exploring tumor reactivity with selected Vδ1TCR for the development of γδT cell-based immunotherapies (20), the absence of functional reactivity by an introduced Vδ1TCR might not necessarily reflect the absence of binding of the Vδ1TCR to its target but rather the lack of a co-stimulation through, e.g., CD8α or other co-stimulatory molecules. In this study, we reported on the capacity of the introduced CD8α co-receptor to successfully redirect non-tumor reactive CD4^+^ TEG011 cells *in vivo* and *in vitro* against tumor targets that express HLA-A*24:02 molecules. We now report on more than 80% of mice showing persistence of CD8-expressing T cells after 4 weeks. TEG011_CD8α cells showed also in absolute numbers higher T cell counts and stable peripheral persistence *in vivo*, which was, however, mainly a consequence of the persistence of CD4^+^CD8^+^ double-positive TEG011_CD8α and not an improved persistence of CD8^+^ single-positive TEG011_CD8α. This finding supports the notions that co-expression of CD4^+^ and CD8^+^ T cells provides an additional survival signal for TEG011 cells. This observation is in line with clinical studies for CD19 CAR T cells that reported that a mixture of both CD4^+^ and CD8^+^ T cells with 1:1 ratio improved tumor remission in B-ALL patients (42, 43). Regardless of the precise underlying molecular mechanism, for the first time we observed tumor clearance in the bone marrow by TEG011_CD8α, but not by TEG011 alone.

Using humanized transgenic mice expressing human HLA-A*24:02, we could study the implication of CD8α introduction to TEG011, referred to as TEG011_CD8α, elucidating their improved efficacy *in vivo*. We provide evidence that TEG011_CD8α effectively cleared tumor cells in bone marrow and elicited better tumor control against human HLA-A*24:02-expressing tumor cells. We cannot entirely exclude that superior tumor control in TEG011_CD8α may have been caused initially by more CD8 single-positive cells in the TEG011_CD8α product compared to TEG011 product, as CD4^+^/CD8^+^ ratios could not be entirely controlled in the experimental setup prior to infusion. However, our mouse model also allowed us to investigate TEG011_CD8α kinetics in the presence of tumor cells; and we observed sustained long-term TEG persistence mainly for γδTCR^+^CD4^+^CD8^+^ double-positive and a decline in γδTCR^+^CD8^+^ single-positive TEG011_CD8α cells. Importantly, the sustained peripheral TEG persistence was only observed for TEG011_CD8α but not TEGLM1_CD8α, highlighting the key role of a functional tumor-reactive γδTCR. This observation rather argues against the classical helper function of γδTCR^+^CD4^+^CD8^+^ double-positive TEG011_CD8α cells within the context of TEG011_CD8α. Hence, the concurrent expression of CD4^+^ and CD8^+^ co-receptor most likely provided additional survival signal for tumor-specific CD4^+^ T cells, which did not, however, translate into classical helper functions towards CD8^+^ T cells (40, 44, 45). CD4^+^ T cells have been reported to avoid expression of inhibitory receptors on CD8^+^ T cells (46) and as an important cell subset to induce memory T cell formation (47). Along this line we observed over time reduced expression of TIM3 in CD8^+^ single-positive TEG011_CD8α cells compared to mock and TEG011 group. CD4^+^CD8^+^ double-positive TEG011_CD8α cells had lower levels of TIM3 when compared to CD8^+^ single-positive TEG011_CD8α cells. These data remain difficult to interpret, and most likely simply reflect different regulation and activation of non-tumor reactive CD4^+^ and tumor-reactive CD8^+^ TEG011 cells, respectively. We also acknowledge that xenograft mouse models do not allow to completely mimic all potential helper roles of human CD4^+^ T cells, due to the lack of human professional antigen-presenting cells.

Reprogramming CD4^+^ T cells by genetic engineering has been reported to clinically impact efficacy and toxicity by high affinity receptors, like CARs (48). Vγ9Vδ2TCR (30) and CD8αβ-independent αβTCRs (32) have been also reported to reprogram CD4^+^ T cells, which not only have the ability to exert tumor cell killing but also induce maturation of professional antigen-presenting cells. Transfer of CD8αβ in combination with intermediate affinity tumor reactive αβTCR has been reported to support tumor control *in vitro* and *in vivo* (49, 50), and for high affinity αβTCR with artificial signaling domains adding CD8α alone has been shown to reprogram CD4^+^ T cells (36). Within this context, our data show that CD8αα in combination with a natural γδTCR serves as costimulatory receptor, as opposed to the well-described inhibitory function of CD8αα on αβT cells within the context of a natural αβTCR. Expression of that CD8αα on activated CD4^+^ and CD8αβ^+^ αβT cells has been reported to act as corepressor by competing with CD8αβ^+^ cells for p56^lck^ signaling molecule (51). Though we investigated the role of CD8αα in the TEG concept, our data support the notion that CD8αα in combination with a γδTCR is synergistic on natural γδT cells, as activated CD8αα^+^ γδT cells were reported in supporting control of HIV infection (52). We have also previously reported significant increases in circulating CD8αα^+^ γδT cells in CMV-positive population (28). Thus, CD8αα appears to have opposing functions on innate and adaptive immune cells, where it acts as costimulatory receptor in the context of a γδTCR.

The precise molecular interaction between CD8αα and its specific ligand in our context remains yet to be unraveled. The CD8αα receptor has been shown to bind to MHC Class I molecules, including HLA-A*02:01, HLA-A*11:01, HLA-B*35:01, HLA-C*07:02, *via* protruding α3 domain loop of MHC molecules with lower affinity than the binding of a TCR-pMHC complex (53–56). Polymorphisms in the MHC α3 domain contributes to a binding variation of CD8αα to different HLA molecules, such as HLA-A*24:02. In this context, HLA-A*24:02 is one of the possible ligands for CD8αα on TEG011, in line with an earlier study that reported CD8αα interaction with HLA-A*24:02 in a similar way with HLA-A*02:01, involving binding to the α2 and α3 domains, as well as to the β2m domain of pMHC complex, but with different conformation that suggests CD8αα plasticity (57). The non-classical MHC molecules are also reported to interact with CD8α, such as HLA-G and HLA-E (58). HLA-G is a known ligand for CD8αα, which is expressed on some colorectal cancer (59–61), while HLA-E is mainly expressed in human endothelial cells and is highly expressed in tumor cells (58). Other studies also demonstrated the interaction between CD8 and CEACAM5, which support the possibility of CEACAM5 as CD8α ligands (62).

Overall, we demonstrate that TEG011 equipped with human CD8α coreceptor elicits superior tumor control and long-term persistence, which mainly impacted numbers of γδTCR^+^CD4^+^CD8^+^ double-positive TEG011_CD8α cells, and associated with better T-cell infiltration. In addition, TEG011_CD8α cells successfully cleared tumor cells in the bone marrow. In contrast to currently emerging immunotherapy approach using CAR T cells, our strategy allows tumor-specific targeting of HLA-A*24:02-positive cancer patients, irrespective of antigen-specific expression on cell surface and the type of cancer, and thus TEG011_CD8α therapy has broader applicability towards a substantial amount of cancer patients with HLA-A*24:02-positive haplotype highlighting its therapeutic potential for further clinical application.

## Data Availability Statement

The original contributions presented in the study are included in the article/**Supplementary Material**. Further inquiries can be directed to the corresponding authors.

## Ethics Statement

The animal study was reviewed and approved by Utrecht Animal Welfare Body (IvD) and Central Authority for Scientific Procedures on Animals (CCD). Written informed consent was obtained from the owners for the participation of their animals in this study.

## Author Contributions

IJ, TS, ZS, and JK conceptualized, designed, and developed the *in vivo* models. IJ, PH, WS, and SH performed the *in vitro* and *in vivo* experiments. LB and AB performed the histopathology examination of the mouse tissues. DB and RO contributed vital components. IJ analyzed all *in vitro* and *in vivo* data and was a major contributor in writing the manuscript. IJ, ZS, and JK interpreted all *in vitro* and *in vivo* data. IJ and JK wrote the manuscript. All authors read, reviewed, and approved the final manuscript.

## Funding

Funding for this study was provided by ZonMW 43400003 and VIDI-ZonMW 917.11.337, KWF 6426, 6790 and 7601 to JK; 12586 to TS and JK; 11393 and 13043 to ZS and JK; 11979 to JK and DB.

## Conflict of Interest

DB, ZS, and JK are inventors on different patents with γδTCR sequences, recognition mechanisms, and isolation strategies. JK is cofounder and shareholder of Gadeta (www.gadeta.nl).

The remaining authors declare that the research was conducted in the absence of any commercial or financial relationships that could be construed as a potential conflict of interest.

## Publisher’s Note

All claims expressed in this article are solely those of the authors and do not necessarily represent those of their affiliated organizations, or those of the publisher, the editors and the reviewers. Any product that may be evaluated in this article, or claim that may be made by its manufacturer, is not guaranteed or endorsed by the publisher.
