# Supplementary material for: Adding Help to an HLA-A*24:02 Tumor-Reactive γδTCR Increases Tumor Control
Source: Front Immunol. 2021 Oct 25;12:752699. doi: 10.3389/fimmu.2021.752699 (PMC8573335; doi:10.3389/fimmu.2021.752699)
Supplement: Supplementary file 1 [file DataSheet_1.docx]

Supplementary Material

# Supplementary Figures and Tables


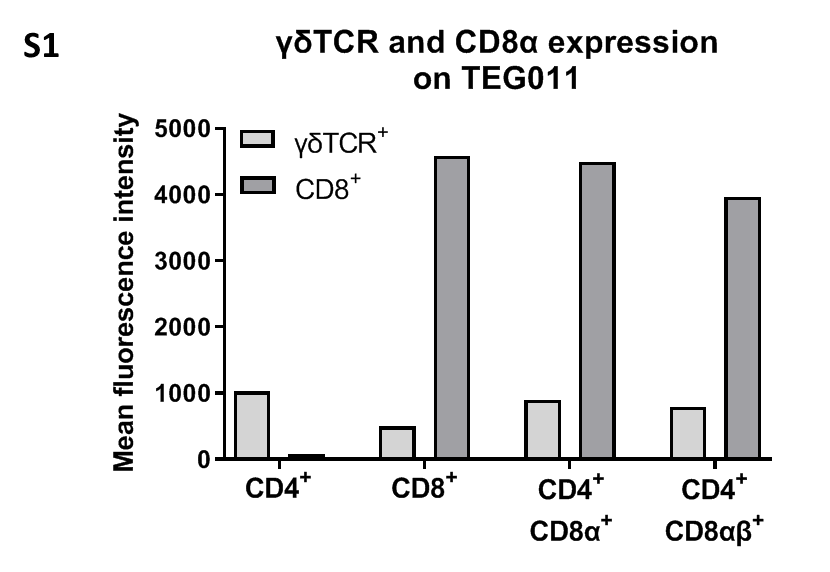


**Figure S1 Transgene expression levels on T cells transduced with the FE11 γδTCR in combination with CD8α alone or CD8α and CD8β.** αβT cells were transduced with the FE11 γδTCR and with either CD8α alone, or CD8α combined with CD8β. Thereafter, CD4^+^, CD8^+^, CD4^+^CD8α^+^ and CD4^+^CD8αβ^+^ TEG011 cells were sorted, and the expression of γδTCR and CD8α was measured by flow cytometry.


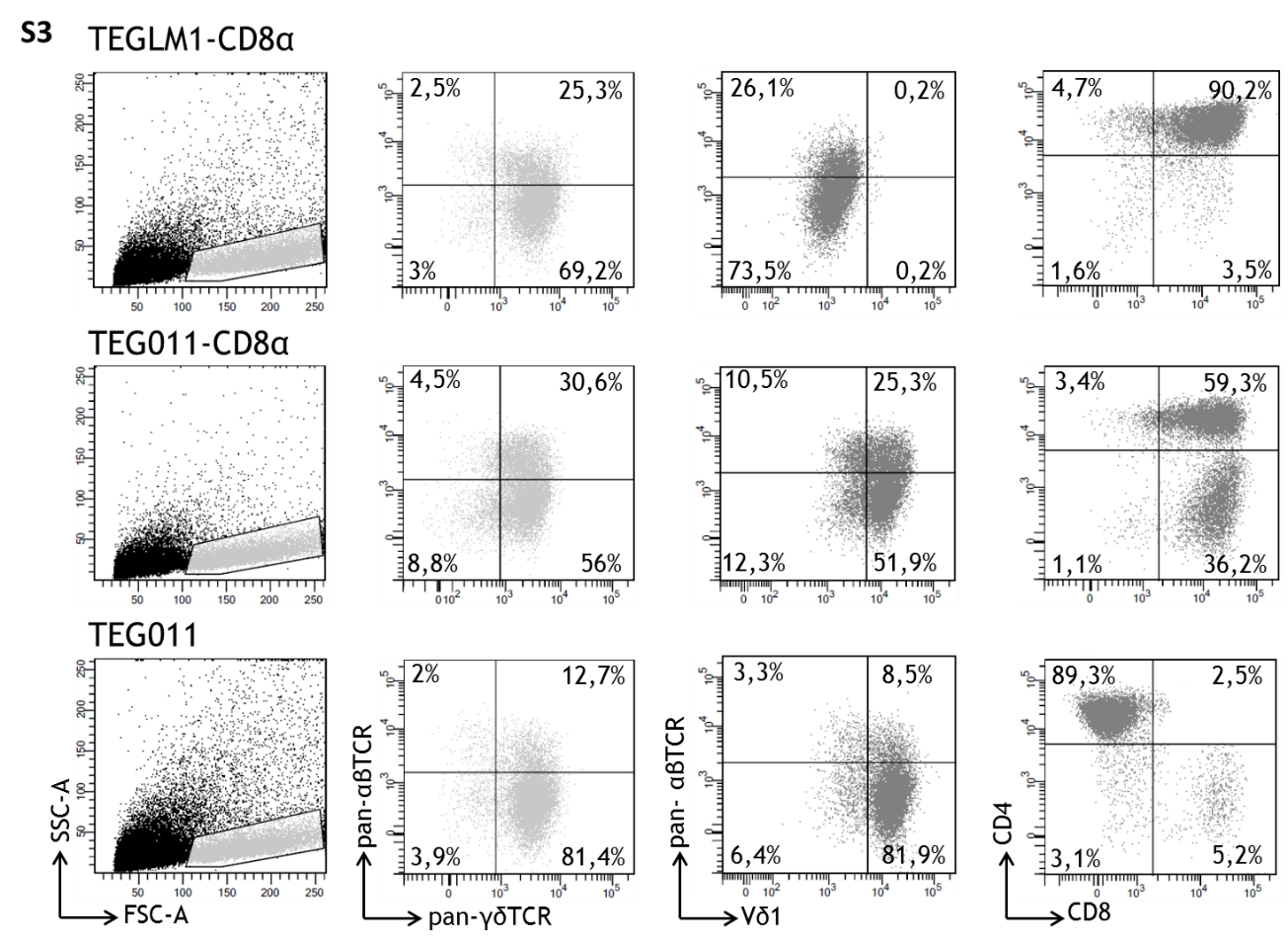


**Figure S2** **γδTCR expression of TEG011, TEG011_CD8a, and TEGLM1_CD8a mock.** Representative flow cytometry plots for γδTCR expression of TEGLM1_CD8α (top panel), TEG011_CD8α (middle panel), and TEG011 (bottom panel) prior to infusion into mice after 2 weeks expansion. Representative plots for Vδ1 TCR expression of TEG011 and TEG011_CD8α were included as a quality control for the flow cytometry panel using pan-γδTCR monoclonal antibody.


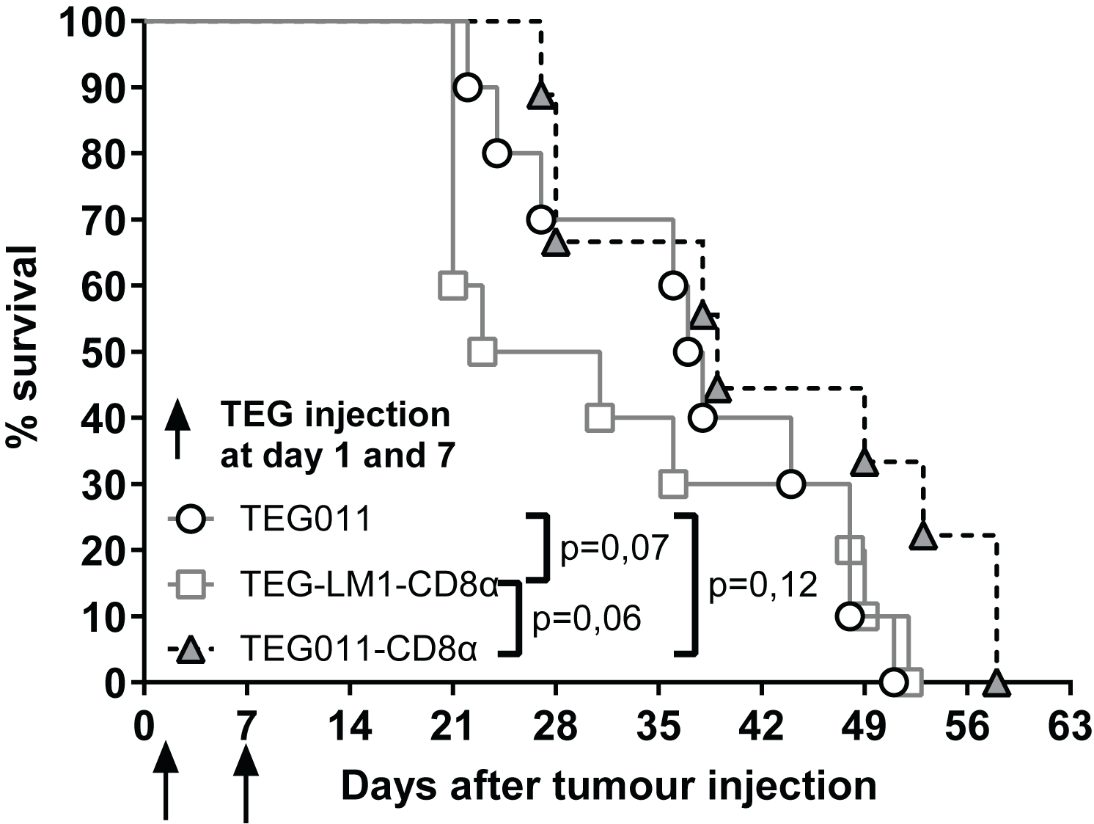


**Figure S3 In vivo antitumor reactivity of TEG011_CD8α in tumor-bearing mice. (A)** Overall survival of treated K562-HLA*A24 luciferase tumor-bearing mice for monitoring efficacy was followed for 60 days. Statistical significances were calculated by log-rank (Mantel-cox) test.


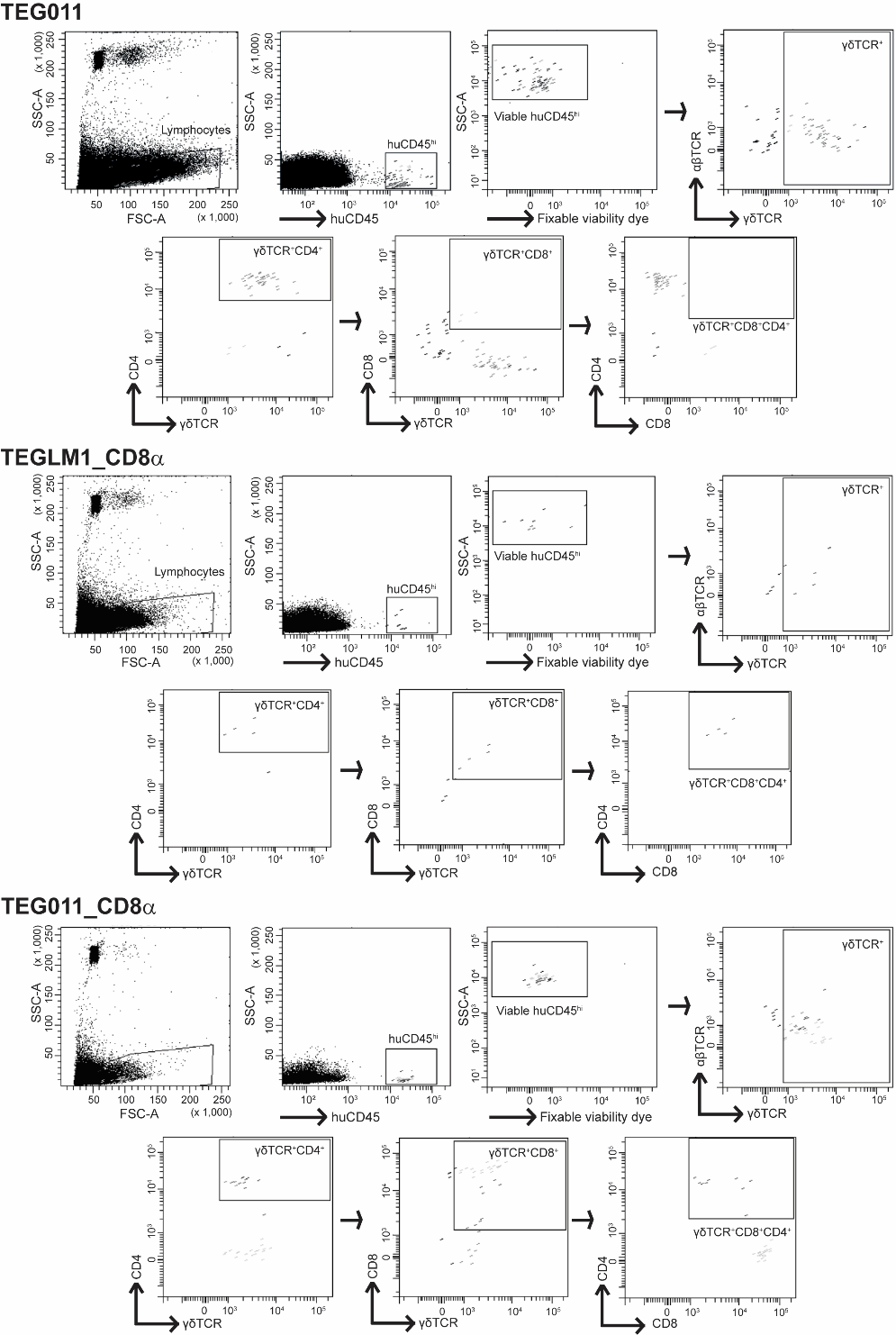


**Figure S4** **Gating strategy for flow cytometry analysis of TEGs persistence in peripheral blood**. (A) Representative flow cytometry plots of peripheral blood in tumor-bearing mice model on Week 2. TEG persistence was measured by quantifying absolute cell number of viable huCD45^+^γδTCR^+^CD8^+^ for TEG011, huCD45^+^γδTCR^+^CD8^+^ single positive and huCD45^+^γδTCR^+^CD4^+^CD8^+^ double positive for TEGLM1_CD8α and TEG011_CD8α mock group.


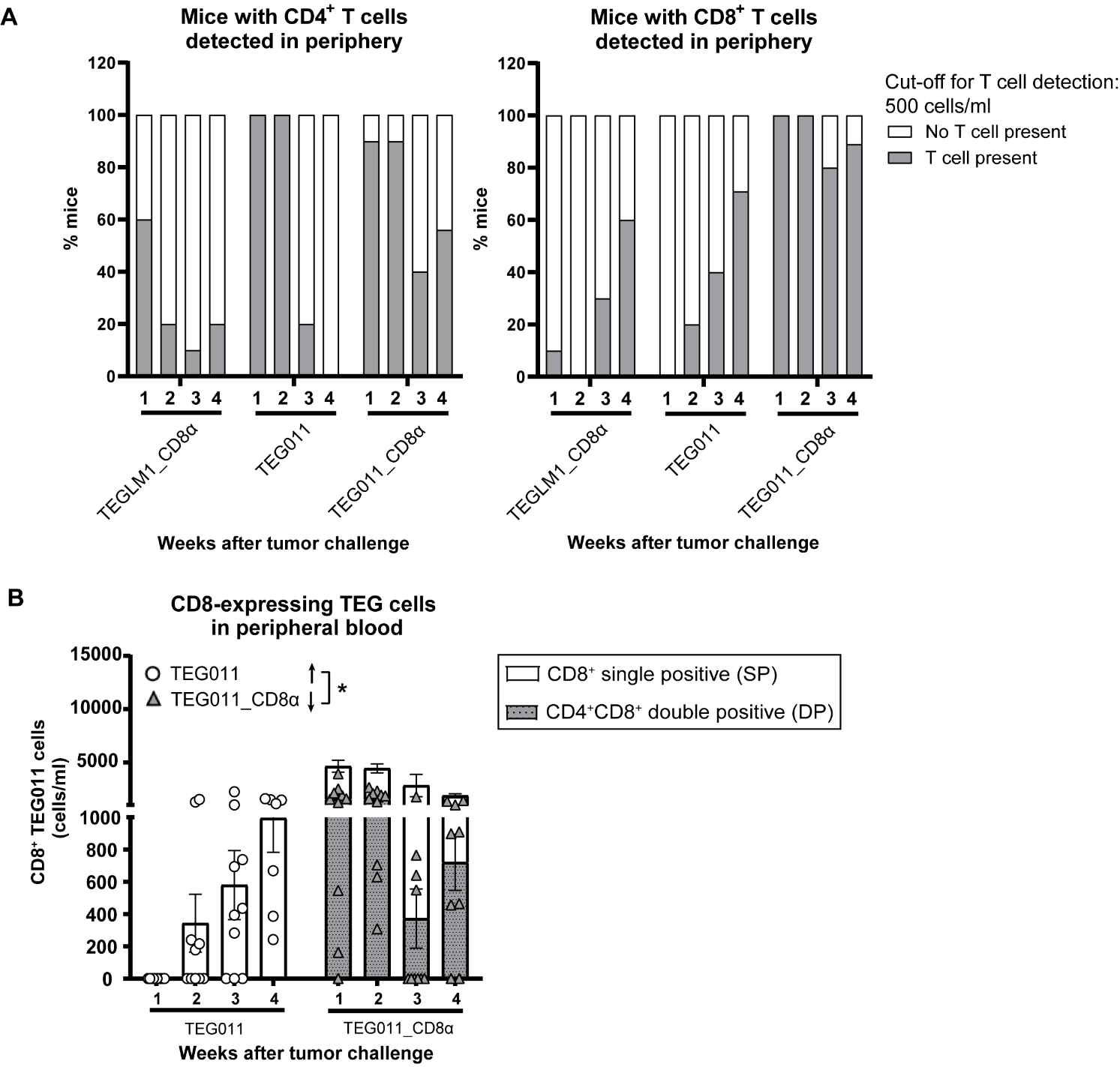


**Figure S5 The presence of T cells observed in peripheral blood of individual mice**. **(A)** Percentage of mice with either CD4^+^ T cells (left panel) or CD8^+^ T cells (right panel) where minimum 500 cells/ml T cells are detected (lower grey bar) or less than 500 cells/ml or no T cells are detected (upper white bar) detected in peripheral blood for each treatment group (n = 10 mice/group). **(B)** Overall persistence of CD8^+^ TEG011 cells in periphery. Absolute number of CD8-expressing TEG011 cells were measured in peripheral blood by flow cytometry for TEG011 (open black circle) and TEG011_CD8α (open black triangle) in tumor-bearing mice. TEG cells are distinguished into two different cellular compartments: CD8^+^ single-positive (SP; white stacked bar) and CD4^+^CD8^+^ double-positive (DP; grey dotted stacked bar). Black arrows indicate higher or lower T cell counts observed. Data represent mean ± SEM of all mice per group (n = 10 mice). Statistical significances were calculated by a mixed-effects model with repeated measures (*P < 0.05).


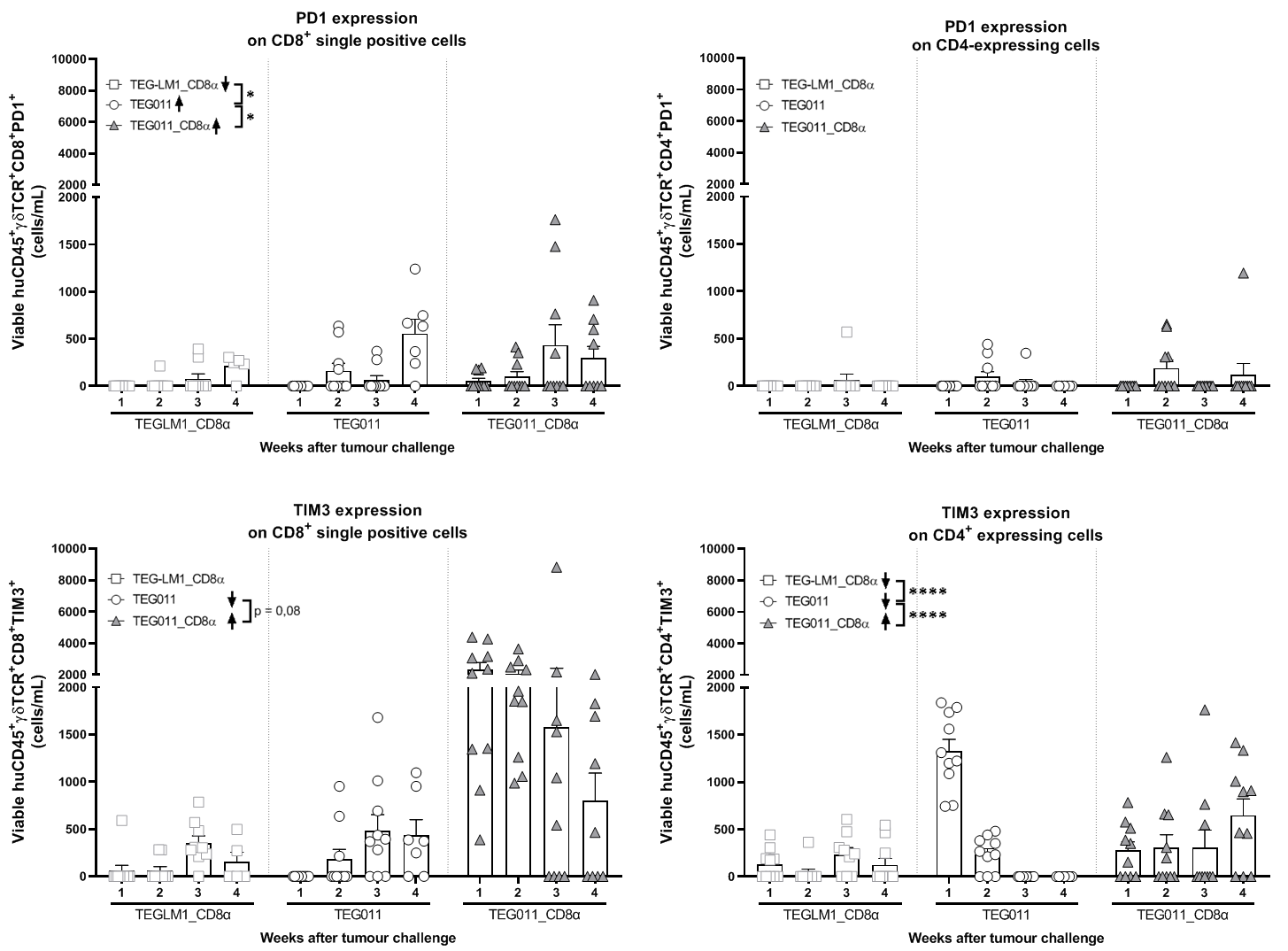


**Figure S6 PD1 and TIM3 expression on CD4^+^ and CD8^+^ TEGs**. Absolute cell counts of PD1-expressing (**A)** and TIM3-expressing **(B)** huCD45^+^γδTCR^+^CD8^+^ single positive cells (left panel) and huCD45^+^γδTCR^+^CD4^+^ single positive or CD4^+^CD8^+^ double positive cells (right panel) were measured by flow cytometry for TEGLM1_CD8α mock (open light gray rectangle), TEG011 (open black circle), and TEG011_CD8α (grey triangle). Black arrows indicate higher or lower T cell counts observed. Data represent mean ± SEM of all mice per group (n = 10 mice). Statistical significances were calculated by a mixed-effects model with repeated measures (*P < 0.05; ****P < 0.0001).

## Supplementary Tables

**Supplementary Table 1. Complete sequence of TEG011_CD8α**

**(pMP71-TCR_FE11γ-T2A-FE11δ_P2A-CD8α)**

>>--TCR FE11γ-->

1081 gctcacttac aggcggccac gcgtggatcc gaattcacc**a tg**ggatgggc tctgctggtg

>---------------------------------TCR FE11γ------------------------------>

1141 ctgctggcct ttctgtctcc tgccagccag aagtccagca acctggaagg cggcaccaag

>----------------------------------TCR FE11γ------------------------------->

1201 agcgtgacca gacctacaag aagcagcgcc gagatcacct gtgacctgac cgtgatcaac

>--------------------------------TCR FE11γ------------------------------>

1261 gccttctaca tccactggta tctgcaccaa gaaggcaagg cccctcagcg gctgctgtac

>----------------------------------TCR FE11γ------------------------------>

1321 tacgatgtgt ccaacagcaa ggacgtgctg gaaagcggac tgagccccgg caagtactac

>---------------------------------TCR FE11γ------------------------------>

1381 acccacacac ctagacggtg gtcctggatc ctgatcctgc ggaacctgat cgagaacgac

>--------------------------------TCR FE11γ------------------------------>

1441 tccggcgtgt actactgcgc cacctgggat agacccgaga tctactataa gaagctgttc

>----------------------------------TCR FE11γ------------------------------->

1501 ggcagcggca ccacactggt ggtcacagac aaacagctgg acgccgacgt gtcccctaag

>--------------------------------TCR FE11γ------------------------------>

1561 cctaccatct tcctgccttc tatcgccgag acaaagctgc agaaggccgg cacctacctg

>---------------------------------TCR FE11γ------------------------------>

1621 tgcctgctgg aaaagttctt cccagacgtg atcaagatcc actgggaaga gaagaagtcc

>----------------------------------TCR FE11γ-------------------------------->

1681 aacaccatcc tgggcagcca agagggcaac accatgaaga ccaacgacac ctacatgaag

>---------------------------------TCR FE11γ------------------------------->

1741 ttcagctggc tgaccgtgcc tgagaagtcc ctggacaaag aacaccggtg catcgtgcgg

>---------------------------------TCR FE11γ------------------------------->

1801 cacgagaaca acaagaacgg cgtggaccaa gagatcatct tcccacctat caagaccgac

>---------------------------------TCR FE11γ------------------------------->

1861 gtcatcacaa tggaccccaa ggacaactgc tccaaggacg ccaacgatac cctgctgctg

>--------------------------------TCR FE11γ----------------------------->

1921 cagctgacaa acaccagcgc ctactacatg tatttgctgc tgttgctgaa gtccgtggtg

>--------------------------------TCR FE11γ------------------------------>

1981 tacttcgcca tcatcacatg ctgcctgctg cggagaaccg ccttctgctg caatggcgag

----->>------------------------------T2A linker------------------------------

2041 aaaagcgtcg acagcggctc tggcagatct ggctctggcg aaggcagagg ctctctgctg

-------------------------T2A linker----------------------->>----------------

2101 acatgtggcg acgtggaaga gaaccccgga cctcgcttaa ttaacatggt gttcagcagc

>---------------------------------TCR FE11δ------------------------------>

2161 ctgctgtgcg tgttcgtggc ctttagctac agcggaagca gcgtggccca gaaagtgaca

>--------------------------------TCR FE11δ------------------------------>

2221 caggcccagt cctccgtgtc tatgcctgtg cggaaagccg tgacactgaa ctgcctgtac

>---------------------------------TCR FE11δ------------------------------>

2281 gagacaagct ggtggtctta ctacatcttc tggtacaagc agctgcccag caaagagatg

>---------------------------------TCR FE11δ------------------------------->

2341 atctttctga tccggcaggg cagcgacgag cagaatgcca agagcggcag atactccgtg

>--------------------------------TCR FE11δ------------------------------>

2401 aacttcaaga aagccgccaa gtctgtggcc ctgaccatct ctgctctgca actggaagat

>---------------------------------TCR FE11δ----------------------------->

2461 agcgccaagt acttctgcgc cctgggcgat tcttatggcg gcggacctct gtacaccgac

>---------------------------------TCR FE11δ------------------------------->

2521 aagctgatct tcggcaaggg caccagagtg accgtggaac ctagaagcca gcctcacacc

>--------------------------------TCR FE11δ------------------------------>

2581 aagccttccg tgtttgtgat gaagaacggc accaacgtgg cctgcctggt caaagagttc

>---------------------------------TCR FE11δ------------------------------>

2641 taccctaagg acatccggat caacctggtg tccagcaaga agatcaccga gttcgacccc

>---------------------------------TCR FE11δ-------------------------------->

2701 gccatcgtga tcagccctag cggcaagtat aacgccgtga agctggggaa gtacgaggac

>---------------------------------TCR FE11δ------------------------------->

2761 agcaatagcg tgacctgcag cgtgcagcat gataacaaga ccgtgcacag caccgatttc

>----------------------------------TCR FE11δ--------------------------------->

2821 gaagtgaaaa ccgactccac cgaccacgtg aagcccaaag agacagagaa caccaagcag

>---------------------------------TCR FE11δ------------------------------->

2881 cccagcaagt cctgccacaa gcctaaggcc atcgtgcaca ccgagaaagt gaacatgatg

>--------------------------------TCR FE11δ------------------------------>

2941 agcctgacag tgctgggcct gagaatgctg ttcgccaaga cagtggccgt gaatttcctg

>-------TCR FE11δ------->>-----------------P2A linker----------------

3001 ctgaccgcca agctgttctt tctgctcgag ggcagcggcg ccacaaattt cagcctgctg

----------------P2A linker--------------------->>----------------------------

3061 aaacaggccg gcgacgtcga agaaaatcct ggaccaatgg ccttaccagt gaccgccttg

>-----------------------------------CD8α------------------------------------->

3121 ctcctgccgc tggccttgct gctccacgcc gccaggccga gccagttccg ggtgtcgccg

>-----------------------------------CD8α------------------------------------->

3181 ctggatcgga cctggaacct gggcgagaca gtggagctga agtgccaggt gctgctgtcc

>-----------------------------------CD8α-------------------------------------->

3241 aacccgacgt cgggctgctc gtggctcttc cagccgcgcg gcgccgccgc cagtcccacc

>-----------------------------------CD8α------------------------------------->

3301 ttcctcctat acctctccca aaacaagccc aaggcggccg aggggctgga cacccagcgg

>-----------------------------------CD8α------------------------------------>

3361 ttctcgggca agaggttggg ggacaccttc gtcctcaccc tgagcgactt ccgccgagag

>-----------------------------------CD8α--------------------------------->

3421 aacgagggct actatttctg ctcggccctg agcaactcca tcatgtactt cagccacttc

>-----------------------------------CD8α-------------------------------------->

3481 gtgccggtct tcctgccagc gaagcccacc acgacgccag cgccgcgacc accaacaccg

------------------------------------CD8α----------------------------------------

3541 gcgcccacca tcgcgtcgca gcccctgtcc ctgcgcccag aggcgtgccg gccagcggcg

>-----------------------------------CD8α------------------------------------->

3601 gggggcgcag tgcacacgag ggggctggac ttcgcctgtg atatctacat ctgggcgccc

>-----------------------------------CD8α-------------------------------->

3661 ctggccggga cttgtggggt ccttctcctg tcactggtta tcacccttta ctgcaaccac

>----------------------------------CD8α------------------------------------->

3721 aggaaccgaa gacgtgtttg caaatgtccc cggcctgtgg tcaaatcggg agacaagccc

>-----------CD8α---------->>

3781 agcctttcgg cgagatacgt ctgata**tga**a aagcttaaca cgagccatag atagaataaa

**Supplementary Table 2. Complete sequence of TEGLM1_CD8α**

**(pMP71-TCRG115γ-T2A-TCRG115δ_LM1_P2A-CD8α)**

>>--TCR G115γ-->

1081 gctcacttac aggcggccac gcgtggatcc gaattcacc**a tg**gtgtccct gctgcacgcc

>--------------------------------TCR G115γ--------------------------------->

1141 agcaccctgg ccgtgctggg cgccctgtgc gtgtatggcg ccggacacct ggaacagccc

>--------------------------------TCR G115γ---------------------------------->

1201 cagatcagca gcaccaagac cctgagcaag accgccaggc tggaatgcgt ggtgtccggc

>--------------------------------TCR G115γ-------------------------------->

1261 atcaccatca gcgccacctc cgtgtactgg tacagagaga gacccggcga ggtcatccag

>--------------------------------TCR G115γ--------------------------------->

1321 ttcctggtgt ccatcagcta cgacggcacc gtgcggaaag agagcggcat ccccagcggc

>--------------------------------TCR G115γ-------------------------------->

1381 aagttcgagg tggacagaat ccccgagacc agcacctcca ccctgaccat ccacaacgtg

>--------------------------------TCR G115γ---------------------------------->

1441 gagaagcagg acatcgccac ctactactgc gccctgtggg aggcccagca ggaactgggc

>--------------------------------TCR G115γ-------------------------------->

1501 aagaaaatca aggtgttcgg ccctggcacc aagctgatca tcaccgacaa gcagctggac

>--------------------------------TCR G115γ-------------------------------->

1561 gccgacgtga gccccaagcc taccatcttc ctgcccagca tcgccgagac caagctgcag

>-------------------------------TCR G115γ-------------------------------->

1621 aaggccggca cctacctgtg cctgctggaa aagttcttcc ccgacgtgat caagatccac

>---------------------------------TCR G115γ--------------------------------->

1681 tgggaggaaa agaagagcaa caccatcctg ggcagccagg aaggcaatac catgaaaacc

>--------------------------------TCR G115γ--------------------------------->

1741 aacgacacct acatgaagtt cagctggctg accgtgcccg agaagagcct ggacaaagag

>--------------------------------TCR G115γ-------------------------------->

1801 cacagatgca tcgtccggca cgagaacaac aagaacggcg tggaccagga aatcatcttc

>--------------------------------TCR G115γ--------------------------------->

1861 ccccccatca agaccgatgt gatcacaatg gaccccaagg acaactgcag caaggacgcc

>------------------------------TCR G115γ------------------------------->

1921 aacgataccc tgctgctgca gctgaccaac accagcgcct actacatgta tctcctgctg

>--------------------------------TCR G115γ------------------------------>

1981 ctgctgaaga gcgtggtgta cttcgccatc atcacctgct gtctgctgcg gcggaccgcc

>------TCR G115γ------->>--------------------T2A linker-------------------

2041 ttctgctgca acggcgagaa gagcgtcgac agcggcagcg ggcgcagcgg cagcggcgaa

------------------------------------T2A linker---------------------------------

2101 ggccgcggca gcctgctgac ctgcggcgat gtggaagaaa accctggccc gcgcttaatt

---->>--------------------------TCR G115δ_LM1------------------------->

2161 aacatggagc ggatcagcag cctgatccac ctgagcctgt tctgggccgg agtgatgagc

>-----------------------------TCR G115δ_LM1----------------------------->

2221 gccatcgagc tggtgcccga gcaccagacc gtgcccgtga gcatcggcgt gcccgccacc

>-----------------------------TCR G115δ_LM1---------------------------->

2281 ctgcggtgca gcatgaaggg cgaggccatc ggcaactact acatcaactg gtacagaaag

>----------------------------TCR G115δ_LM1---------------------------->

2341 acccagggca acaccatgac cttcatctac cgggagaagg acatctacgg ccctggcttc

>-----------------------------TCR G115δ_LM1---------------------------->

2401 aaggacaact tccagggcga catcgacatc gccaagaacc tggccgtgct gaagatcctg

>-----------------------------TCR G115δ_LM1------------------------------>

2461 gcccccagcg agagggacga gggcagctac tactgcgcct gcgacaccct ggccaccgac

>------------------------------TCR G115δ_LM1----------------------------->

2521 aagctgatct tcggcaaggg cacccgggtg accgtggagc ccagaagcca gccccacacc

>----------------------------TCR G115δ_LM1---------------------------->

2581 aagcccagcg tgttcgtgat gaagaacggc accaacgtgg cctgcctggt gaaagagttc

>----------------------------TCR G115δ_LM1---------------------------->

2641 taccccaagg acatccggat caacctggtg tccagcaaga agatcaccga gttcgacccc

>-----------------------------TCR G115δ_LM1----------------------------->

2701 gccatcgtga tcagccccag cggcaagtac aacgccgtga agctgggcaa gtacgaggac

>-----------------------------TCR G115δ_LM1----------------------------->

2761 agcaacagcg tgacctgcag cgtgcagcac gacaacaaga ccgtgcacag caccgacttc

>-----------------------------TCR G115δ_LM1------------------------------>

2821 gaggtgaaaa ccgactccac cgaccacgtg aagcccaaag agaccgagaa caccaagcag

>-----------------------------TCR G115δ_LM1----------------------------->

2881 cccagcaaga gctgccacaa gcccaaggcc atcgtgcaca ccgagaaggt gaacatgatg

>----------------------------TCR G115δ_LM1--------------------------->

2941 agcctgaccg tgctgggcct gcggatgctg ttcgccaaga cagtggccgt gaacttcctg

>----TCR G115δ_LM1--->>----------------P2A linker----------------

3001 ctgaccgcca agctgttctt cctgctcgag ggcagcggcg ccacaaattt cagcctgctg

-------------------P2A linker------------------>>----------CD8α---------->

3061 aaacaggccg gcgacgtcga agaaaatcct ggaccaatgg ccttaccagt gaccgccttg

>------------------------------------CD8α----------------------------------->

3121 ctcctgccgc tggccttgct gctccacgcc gccaggccga gccagttccg ggtgtcgccg

>-------------------------------------CD8α----------------------------------->

3181 ctggatcgga cctggaacct gggcgagaca gtggagctga agtgccaggt gctgctgtcc

>------------------------------------CD8α------------------------------------>

3241 aacccgacgt cgggctgctc gtggctcttc cagccgcgcg gcgccgccgc cagtcccacc

>-------------------------------------CD8α----------------------------------->

3301 ttcctcctat acctctccca aaacaagccc aaggcggccg aggggctgga cacccagcgg

>-----------------------------------CD8α----------------------------------->

3361 ttctcgggca agaggttggg ggacaccttc gtcctcaccc tgagcgactt ccgccgagag

>----------------------------------CD8α--------------------------------->

3421 aacgagggct actatttctg ctcggccctg agcaactcca tcatgtactt cagccacttc

>-------------------------------------CD8α------------------------------------>

3481 gtgccggtct tcctgccagc gaagcccacc acgacgccag cgccgcgacc accaacaccg

>--------------------------------------CD8α------------------------------------>

3541 gcgcccacca tcgcgtcgca gcccctgtcc ctgcgcccag aggcgtgccg gccagcggcg

>------------------------------------CD8α------------------------------------>

3601 gggggcgcag tgcacacgag ggggctggac ttcgcctgtg atatctacat ctgggcgccc

>----------------------------------CD8α--------------------------------->

3661 ctggccggga cttgtggggt ccttctcctg tcactggtta tcacccttta ctgcaaccac

>------------------------------------CD8α----------------------------------->

3721 aggaaccgaa gacgtgtttg caaatgtccc cggcctgtgg tcaaatcggg agacaagccc

>------------CD8α---------->>

3781 agcctttcgg cgagatacgt ctgata**tga**a aagcttaaca cgagccatag atagaataaa
